# *Paenibacillus assamensis* in Joint Fluid of Man with Suspected Tularemia, China

**DOI:** 10.3201/eid2408.180260

**Published:** 2018-08

**Authors:** Enmin Zhang, Hongchao Lu, Qiuhong Liu, Zhigang Tang, Duanjun Li, Lin Jiang, Qisheng He, Niu Pan, Yanhua Wang

**Affiliations:** Chinese Center for Disease Control and Prevention, Beijing, China (E. Zhang, Y. Wang);; Center for Disease Prevention and Control of Qianxinan Prefecture of Guizhou Province, Xingyi, China (H. Lu, Q. Liu, Z. Tang, L. Jiang, Q. He, N. Pan);; Hospital of Traditional Chinese Medicine of Qianxinan Prefecture, Xingyi (D. Li)

**Keywords:** Paenibacillus, Francisella tularensis, tularemia, knee joint, patients, China, bacteria

## Abstract

*Paenibacillus assamensis* is a bacterium usually found in warm springs. We detected *P. assamensis* in a man with suspected tularemia. The strain isolated from the man’s knee joint fluid was identified as *P. assamensis* after analysis of a homologous sequence of the 16S rRNA gene.

The genus *Paenibacillus* comprises >89 species. Some of these are pathogens in honeybees or other invertebrates; others are occasional opportunistic pathogens in humans (*1*,*2*). Bacteria belonging to the genus *Paenibacillus* have been isolated from various environments, such as soil, water, rhizospheres, vegetable matter, forage, and insect larvae, as well as from clinical samples (*3*). Tularemia, caused by the gram-negative intracellular pathogen *Francisella tularensis*, is highly virulent in humans and animals. An isolate from the joint fluid of a man in China in whom suspected tularemia was diagnosed recently was identified as *Paenibacillus assamensis*, a species usually found in warm springs.

A 44-year-old farmer was admitted to the surgical department of the Hospital of Traditional Chinese Medicine of Qianxinan Prefecture (Xingyi, China) on June 28, 2016. He had swelling and aching in his left knee that had appeared without an obvious cause and lasted for ≈6 months (Figure, panel A). He denied being bitten by a mosquito or other insect. Moreover, he had no contact with any animal before onset. In January 2016, he was admitted to the local county hospital for fever, cough, and pectoralgia. An antituberculosis regimen was started 3 days later. After he took the prescribed medicine for 15 days, his left knee began to swell and ache, accompanied by limitation of movement. However, no improvement was evident after drug withdrawal.

**Figure:**
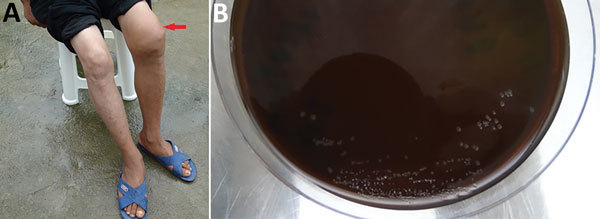
Patient from whom *Paenibacillus assamensis* was isolated from knee joint fluid, China, 2016. A) Left knee joint of the patient was obviously swollen (red arrow). B) *P. assamensis* isolated from the patient’s articular fluid appeared on chocolatized erythrocytes.

His body temperature fluctuated from 36.0°C to 36.8°C. A semisolid mass of 9.7 × 2.1 cm^2^ was detected on ultrasound examination 0.4 cm from the left popliteal fossa to the subcutaneous surface. No acid-resistant bacilli were detected in the articular puncture fluid, which was inoculated using blood plate media. Gray migratory colonies of gram-negative bacteria appeared after 2 days. The presumptive identification of the isolate using the gram-negative card on the VITEK 2 Compact System (bioMérieux, St. Louis, MO, USA) was *F. tularensis*. A Cystine heart agar enriched with chocolatized erythrocyte medium was inoculated with the isolate, and gray opaque colonies ≈1 mm in diameter emerged after 2 days (Figure, panel B). The latex-agglutination test showed that the fresh strain and the patient’s serum were negative for *F. tularensis*–specific antigens and antibodies.

We amplified the 16S rRNA gene of the bacterial genomic DNA using 2 universal bacterial primers, 27f and 1492r (*4*). Next, a 1,379-nt continuous stretch (GenBank accession no. MG847149) was obtained by 2-way sequencing of high-quality amplicons, which we used as a query to search for homologous sequences in the GenBank database. The query coverage was 100%, and the highest consistent sequence was that of *P. assamensis* strain GPTSA 11 16S rRNA gene. We further amplified the 16S rRNA gene of the patient’s DNA using another pair of primers, 8–27f and 1500r (*5*). The highest consistent sequence obtained this time was 1,429 nt (GenBank accession number MG847150), still in the 16S rRNA gene of *P. assamensis* strain GPTSA 11. The analysis of the sequences indicated that the 2 amplicons contained the consensus signature sequence stretches PAEN 515F (*6*) and PAEN 862F (*7*), which are mostly found among different species within the genus *Paenibacillus*.

The local hospital detected *F. tularensis* in the patient with suspected tularemia on the basis of PyrA-positive results using the VITEK 2 Compact System. No recent studies have reported the association between PyrA and *F. tularensis*. The biochemical tests also showed that the strain could not ferment glucose and maltose. However, *F. tularensis* type A and type B are capable of fermenting glucose and maltose (*8*). Moreover, *F. tularensis*–specific antigen and antibody tests did not confirm that this strain was *F. tularensis* (*9*).

Both amplicons of the patient’s 16S rRNA gene contained PAEN 515F and PAEN 862F signature sequences. After searching the homologous sequence of the 2 amplicons, the 16S rRNA gene sequence of *P. assamensis* GPTSA 11 displayed higher homology. Therefore, we concluded that the bacterium isolated from the patient’s joint fluid was not *F. tularensis* but *P. assamensis*. 

In 2005, a new species of *Paenibacillus* named *P. assamensis* GPTSA 11 was isolated from a warm spring in Assam, India (*10*). Since then, *P. assamensis* had not been isolated from other environments or patients. Our findings emphasize the need to consider new approaches for preventing infection in the environments where *P. assamensis* exists.

This patient remained at home to recuperate because of his obscure symptoms and financial constraints, but his symptoms did not abate until the follow-up in July 2017. He was advised to return to the hospital for treatment with drugs targeting *P. assamensis*. Isolation of *P. assamensis* from the living and working environments of patients, including soil and water, can successfully reveal the source of infection.

## References

[1] Reboli AC, Bryan CS, Farrar WE. Bacteremia and infection of a hip prosthesis caused by *Bacillus alvei.* J Clin Microbiol. 1989;27:1395–6. 2754007 10.1128/jcm.27.6.1395-1396.1989 PMC267567

[2] Ko KS, Kim YS, Lee MY, Shin SY, Jung DS, Peck KR, et al. *Paenibacillus konsidensis* sp. nov., isolated from a patient. Int J Syst Evol Microbiol. 2008;58:2164–8. 10.1099/ijs.0.65534-0 18768623

[3] Grady EN, MacDonald J, Liu L, Richman A, Yuan ZC. Current knowledge and perspectives of *Paenibacillus*: a review. Microb Cell Fact. 2016;15:203. 10.1186/s12934-016-0603-7 27905924 PMC5134293

[4] Lane DJ. 16S/23S rRNA sequencing. In: Stackebrandt E, Goodfellow M, editors. Nucleic acid techniques in bacterial systematics. New York: John Wiley & Sons, Inc.; 1991. p. 115–76.

[5] Pandey KK, Mayilraj S, Chakrabarti T. *Pseudomonas indica* sp. nov., a novel butane-utilizing species. Int J Syst Evol Microbiol. 2002;52:1559–67. 12361258 10.1099/00207713-52-5-1559

[6] Shida O, Takagi H, Kadowaki K, Nakamura LK, Komagata K. Transfer of *Bacillus alginolyticus, Bacillus chondroitinus, Bacillus curdlanolyticus, Bacillus glucanolyticus, Bacillus kobensis*, and *Bacillus thiaminolyticus* to the genus *Paenibacillus* and emended description of the genus *Paenibacillus.* Int J Syst Bacteriol. 1997;47:289–98. 10.1099/00207713-47-2-289 9103612

[7] Pettersson B, Rippere KE, Yousten AA, Priest FG. Transfer of *Bacillus lentimorbus* and *Bacillus popilliae* to the genus *Paenibacillus* with emended descriptions of *Paenibacillus lentimorbus* comb. nov. and *Paenibacillus popilliae* comb. nov. Int J Syst Bacteriol. 1999;49:531–40. 10.1099/00207713-49-2-531 10319474

[8] Chu MC, Weyant R. *Francisella* and *Brucella*. In: Murray PR, editor. Manual of clinical microbiology. 8th ed. Washington (DC): American Society for Microbiology; 2003. p. 789–97.

[9] World Health Organization. WHO guidelines on tularaemia. Geneva: The Organization; 2007.

[10] Saha P, Mondal AK, Mayilraj S, Krishnamurthi S, Bhattacharya A, Chakrabarti T. *Paenibacillus assamensis* sp. nov., a novel bacterium isolated from a warm spring in Assam, India. Int J Syst Evol Microbiol. 2005;55:2577–81. 10.1099/ijs.0.63846-0 16280530

